# Supplementary material for: The direct and indirect effects of community beliefs and attitudes on postpartum contraceptive method choice among young women ages 15–24 in Nigeria
Source: PLoS One. 2022 Jan 27;17(1):e0261701. doi: 10.1371/journal.pone.0261701 (PMC8794167; doi:10.1371/journal.pone.0261701)
Supplement: S1 File — (DOCX) [file pone.0261701.s001.docx]

**Supporting information**

In the paper text and in Tables 3a and 3b, the results from the joint estimation of our three outcome equations that are corrected for selection and the presence of endogenous regressors are shown. For purposes of comparison, we show in this supporting information file the uncorrected results in Appendix Tables 1a and 1b. This file also contains the estimation results for the heterogeneity parameters that are strongly significant, indicating the need to use a joint estimation strategy for the three outcome equations.

**Estimation of the Unobserved Heterogeneity Distribution**

Equations (1) – (3) are estimated jointly to control for selection and the presence of endogenous regressors. Joint estimation typically requires one to make distributional assumptions about the error terms -- for example, multivariate normality is often assumed. Rather than make specific parametric assumptions about the distribution of the $\mu's$ and $\varepsilon's$ in equations (1) –(3),we use a variation of the discrete factor approximation [38]. Specifically, we use what Mroz [49] refers to as non-linear heterogeneity where mass points are estimated for each equation along with a common set of probabilities. This form for the discrete factor model allows for very general patterns of correlation and has been shown to work very well in Monte Carlo experiments [41, 49]. The corrected model estimates 21 heterogeneity parameters and probability weights that are presented in Appendix Table 2 below. Specifically, we estimated three mass points for the community level error ($\mu$) and two mass points for the individual level error ($\varepsilon$) for each of the error terms in each equation (1)-(3) in the main text along with two sets of common probabilities. A chi squared test of the null hypothesis that these estimated parameters are jointly zero yields a chi squared statistic of 158.10 which has a p value of essentially zero. Therefore, there is strong evidence of the need to correct for unobserved heterogeneity.

**Simulation methodology**

The simulation works in the following manner. We used the estimated coefficients, mass points and probability weights to predict the probability of whether or not woman i from community j had first sex at age 12. We then compared this predicted probability to a random draw from a uniform distribution with endpoints zero and one. If, for example, the predicted probability of first sex was 0.05, we would assign first sex at age 12 to the woman if the uniform random variable was between 0 and 0.05. If the woman was not assigned first sex at age 12, we incremented her age by one year and repeated the procedure. Once the woman was assigned an age at first sex, we then proceed to the birth equation and use a similar procedure to determine age at each birth and the total number of births by age 24. Finally, following each birth, we predict the probability that the woman uses no method in the year following the birth or one of our four method categories.

This process was followed for each woman in the sample; in the tables we report averages for the sample of 3,454 respondents. In the simulations, we varied the community level variables one at a time and then determined age at first sex, the timing and number of births to each woman up to age 24 and contraceptive method choice. All other explanatory variables were kept at their actual values for each woman so that we could isolate the effects of interest. Finally, we used a parametric bootstrap procedure to calculate standard errors for all predictions using 1,000 bootstrap replications.

| **Uncorrected multivariate results** | | | | | | | | | | | | | | | | | | | | | | | | | |  |  |  |  |  |  |  |
| --- | --- | --- | --- | --- | --- | --- | --- | --- | --- | --- | --- | --- | --- | --- | --- | --- | --- | --- | --- | --- | --- | --- | --- | --- | --- | --- | --- | --- | --- | --- | --- | --- |
| **Appendix Table 1a. Multivariate results for postpartum contraceptive method choice (Table 3a) and the timing of age at first sex and timing of births (Table 3b) from uncorrected models, Nigeria, 2017** | | | | | | | | | | | | | | | | | | | | | | | | | |  |  |  |  |  |  |  |
|  | **Method: Traditional vs. Non-use** | | | | | |  | | **Method: LAM vs. Non-use** | | | | | |  | | **Method: Shop/pharmacy-based method vs.**  **Non-use** | | | | | |  | | **Method: Facility-based method vs.**  **Non-use** | | | | | | |  |
|  | Uncorrected Results | | | | | |  | | Uncorrected Results | | | | | |  | | Uncorrected Results | | | | | |  | | Uncorrected Results | | | | |  |  |  |
|  | Coef. | | SE | | z | |  | | Coef. | | SE | | z | |  | | Coef. | | SE | | z | |  | | Coef. | | SE | | z | | | |
| **Individual Variables** |  | |  | |  | |  | |  | |  | |  | |  | |  | |  | |  | |  | |  | |  | |  | | | |
| Constant | -5.700 | 1.34 | | -4.24*** | |  | | -1.726 | | 1.88 | | -0.92 | |  | | -6.914 | | 1.54 | | -4.50*** | |  | | -3.798 | | 1.28 | | -2.96** | | |  |  |
| Age | 0.088 | 0.07 | | 1.19 | |  | | 0.140 | | 0.09 | | 1.53 | |  | | 0.156 | | 0.09 | | 1.71† | |  | | -0.017 | | 0.09 | | -0.19 | | |  |  |
| Education | 0.107 | 0.04 | | 2.84** | |  | | 0.034 | | 0.05 | | 0.67 | |  | | 0.080 | | 0.03 | | 2.35* | |  | | 0.032 | | 0.03 | | 1.15 | | |  |  |
| Ilorin (ref. Kaduna) | 0.516 | 0.40 | | 1.28 | |  | | -3.207 | | 1.24 | | -2.58** | |  | | 1.282 | | 0.41 | | 3.11** | |  | | -0.648 | | 0.46 | | -1.41 | | |  |  |
| Jos (ref. Kaduna) | 0.818 | 0.37 | | 2.20* | |  | | -0.489 | | 0.48 | | -1.01 | |  | | 0.483 | | 0.41 | | 1.19 | |  | | 0.277 | | 0.33 | | 0.84 | | |  |  |
| Muslim (ref. non-Muslim) | 0.351 | 0.32 | | 1.09 | |  | | -0.941 | | 0.32 | | -2.90** | |  | | -0.654 | | 0.36 | | -1.82† | |  | | -0.751 | | 0.29 | | -2.60* | | |  |  |
| Religiosity (ref. not religious) | 0.046 | 0.30 | | 0.15 | |  | | 0.146 | | 0.45 | | 0.32 | |  | | 0.012 | | 0.30 | | 0.04 | |  | | -0.076 | | 0.26 | | -0.29 | | |  |  |
| Lived in city ≤1 yr. (ref. >5 yrs) | -0.077 | 0.41 | | -0.19 | |  | | 0.701 | | 0.62 | | 1.14 | |  | | -0.175 | | 0.49 | | -0.36 | |  | | -0.374 | | 0.45 | | -0.82 | | |  |  |
| Lived in city 1-5 yrs (ref. >5 yrs) | 0.094 | 0.37 | | 0.25 | |  | | 0.507 | | 0.51 | | 1.00 | |  | | 0.608 | | 0.33 | | 1.85† | |  | | 0.245 | | 0.28 | | 0.88 | | |  |  |
| 1^st^ birth (ref. 3+ order birth) | -0.014 | 0.44 | | -0.03 | |  | | -0.782 | | 0.45 | | -1.74† | |  | | -0.211 | | 0.58 | | -0.36 | |  | | -1.921 | | 0.44 | | -4.36*** | | |  |  |
| 2^nd^ birth (ref. 3+ order birth) | 0.267 | 0.36 | | 0.75 | |  | | -0.603 | | 0.46 | | -1.32 | |  | | -0.216 | | 0.50 | | -0.43 | |  | | -0.907 | | 0.32 | | -2.88** | | |  |  |
| Age at first sex | 0.034 | 0.08 | | 0.42 | |  | | -0.139 | | 0.10 | | -1.44 | |  | | 0.072 | | 0.09 | | 0.76 | |  | | 0.238 | | 0.09 | | 2.70** | | |  |  |
| **Community Variable** |  |  | |  | |  | |  | |  | |  | |  | |  | |  | |  | |  | |  | |  | |  | | |  |  |
| Myths Norm | -0.245 | 0.20 | | -1.25 | |  | | -0.239 | | 0.37 | | -0.65 | |  | | -0.168 | | 0.21 | | -0.80 | |  | | -0.355 | | 0.18 | | -1.99* | | |  |  |
| Contraceptive Norm | -0.155 | 0.24 | | -0.65 | |  | | -0.069 | | 0.30 | | -0.23 | |  | | 0.563 | | 0.20 | | 2.77** | |  | | 0.670 | | 0.21 | | 3.16** | | |  |  |
| †p < .10; *p < .05; **p < .01; ***p<0.001. Each woman may contribute multiple observations based on her sexual experience, birth experience and postpartum contraceptive use patterns. | | | | | | | | | | | | | | | | | | | | | | | | | |  |  |  |  |  |  |  |

**Appendix Table 1b. Multivariate results for postpartum contraceptive method choice (Table 3a) and the timing of age at first sex and timing of births (Table 3b) from uncorrected models, Nigeria, 2017**

|  | **Timing of Age at First Sex** | | | **Timing of Births** | | |
| --- | --- | --- | --- | --- | --- | --- |
|  | Uncorrected Results | | | Uncorrected Results | | |
| **Individual Variables** | Coef. | SE | z | Coef. | SE | z |
| Constant | -25.526 | 1.25 | -20.50*** | -22.562 | 1.68 | -13.45*** |
| Age | 2.391 | 0.14 | 16.97*** | 2.308 | 0.17 | 13.52*** |
| Age Squared | -0.056 | 0.00 | -14.18*** | -0.052 | 0.00 | -11.84*** |
| Education | -0.145 | 0.02 | -8.62*** | -0.042 | 0.01 | -3.82*** |
| Ilorin (ref. Kaduna) | 0.173 | 0.12 | 1.39 | -0.278 | 0.10 | -2.81** |
| Jos (ref. Kaduna) | 0.125 | 0.12 | 1.05 | -0.132 | 0.09 | -1.41 |
| Muslim (ref. non-Muslim) | -0.272 | 0.10 | -2.69** | 0.778 | 0.11 | 7.25*** |
| Religiosity (ref. not religious) | 0.224 | 0.08 | 2.73** | -0.207 | 0.09 | -2.35* |
| Lived in city ≤1 yr. (ref. >5 years) | 0.226 | 0.11 | 2.11* | -0.550 | 0.13 | -4.27*** |
| Lived in city 1-5 yrs (ref. >5 years) | 0.410 | 0.09 | 4.48*** | 0.119 | 0.08 | 1.43 |
| Age at first sex | na | na | na | -0.245 | 0.01 | -16.83*** |
| **Community Variables** |  |  |  |  |  |  |
| Childbearing Norm | na | na | na | 0.454 | 0.17 | 2.64** |
| All or most unmarried girls sexually active | 0.709 | 0.24 | 2.97** | na | na | na |

†p < .10; *p < .05; **p < .01; ***p<0.001. Each woman may contribute multiple observations based on her sexual experience, birth experience and postpartum contraceptive use patterns.

| **Appendix Table 2. Heterogeneity parameter estimates for the corrected model^a^** | | | | | | | |
| --- | --- | --- | --- | --- | --- | --- | --- |
|  | **Coef.** | **SE** | **z** |  |  | **Model Common Probability Weights** | |
| Timing of First Sex | | | | |  |  |  |
| Community 1 | 0 |  |  |  |  | Community 1 | 0.365 |
| Community 2 | 1.638 | 0.19 | 8.41*** |  |  | Community 2 | 0.073 |
| Community 3 | 0.943 | 0.16 | 6.05*** |  |  | Community 3 | 0.562 |
| Individual 1 | 0 |  |  |  |  | Individual 1 | 0.583 |
| Individual 2 | 2.10 | 0.35 | 5.93*** |  |  | Individual 2 | 0.417 |
| Birth Hazard | | | | |  |  |  |
| Community 1 | 0 |  |  |  |  |  |  |
| Community 2 | 0.009 | 0.15 | 0.06 |  |  |  |  |
| Community 3 | 0.172 | 0.11 | 1.64 |  |  |  |  |
| Individual 1 | 0 |  |  |  |  |  |  |
| Individual 2 | 0.202 | 0.12 | 1.75† |  |  |  |  |
| Method Choice | | | | |  |  |  |
| Traditional versus No Method | | | | | | | |
| Community 1 | 0 |  |  |  |  |  |  |
| Community 2 | -0.603 | 0.83 | -0.73 |  |  |  |  |
| Community 3 | 0.696 | 0.48 | 1.45 |  |  |  |  |
| Individual 1 | 0 |  |  |  |  |  |  |
| Individual 2 | 1.437 | 1.17 | 1.22 |  |  |  |  |
| Lam versus No Method | | | | | | | |
| Community 1 | 0 |  |  |  |  |  |  |
| Community 2 | -1.223 | 0.87 | -1.41 |  |  |  |  |
| Community 3 | 2.878 | 1.02 | 2.82** |  |  |  |  |
| Individual 1 | 0 |  |  |  |  |  |  |
| Individual 2 | -6.005 | 1.44 | 4.18*** |  |  |  |  |
| Short-Acting versus No Method | | | | | | | |
| Community 1 | 0 |  |  |  |  |  |  |
| Community 2 | -1.950 | 0.82 | -2.38* |  |  |  |  |
| Community 3 | 0.985 | 0.75 | 1.31 |  |  |  |  |
| Individual 1 | 0 |  |  |  |  |  |  |
| Individual 2 | -2.411 | 1.16 | -2.07* |  |  |  |  |
| Long-Acting versus No Method | | | | | | | |
| Community 1 | 0 |  |  |  |  |  |  |
| Community 2 | -0.360 | 0.53 | -0.68 |  |  |  |  |
| Community 3 | -0.178 | 0.35 | -0.51 |  |  |  |  |
| Individual 1 | 0 |  |  |  |  |  |  |
| Individual 2 | 0.811 | 1.64 | 0.49 |  | | | |

^a^See equations (1)-(3) for an explanation of these parameters. †p < .10; *p < .05; **p < .01; ***p<0.001.

**References**

49. Mroz TA. Discrete factor approximations in simultaneous equation models: Estimating the impact of a dummy endogenous variable on a continuous outcome. Journal of Econometrics. 1999; 92(2): 233–274.
