# Supplementary material for: The direct and indirect effects of community beliefs and attitudes on postpartum contraceptive method choice among young women ages 15–24 in Nigeria
Source: PLoS One. 2022 Jan 27;17(1):e0261701. doi: 10.1371/journal.pone.0261701 (PMC8794167; doi:10.1371/journal.pone.0261701)
Supplement: S2 File — (DOCX) [file pone.0261701.s002.docx]

**Supporting information**

The simulation Fortran program used for this study was written by one of the co-authors of this article (DKG) and was specifically written to do the simulations described in the text. This supporting information file includes a copy of the manual for one of the estimation approaches used and an example of the setup file as well as information on the actual simulation program (simulation.f).

**Manual for LEO (Discrete Factor Approximation Method)**

**Leo**

INTRODUCTION

LEO is a Fortran-based program that jointly estimates multiple logit, mlogit, conditional logit, mixed logit and/or continuous outcome equations using full-information maximum likelihood. LEO uses discrete factors (the mass point method) to allow both community (cluster) and individual level correlation between the error terms. LEO maximizes the likelihood function using the Goldfeld-Quandt non-linear optimization package (www.quandt.com/gqopt.html) that is marketed by Richard Quandt from Princeton University. The package includes a large number of non-linear optimization packages. The DFAM (LEO) program can invoke two of the packages: the Davidon-Fletcher-Powell (DFP) algorithm and the quadratic hill climbing algorithm (GRADX). A common strategy is to use DFP as a first step and then switch to GRADX when one is close to the maximum of the likelihood function to get more precision and a better estimate of the covariance matrix of the parameter estimates. LEO uses the default versions of the algorithms.

The following likelihood function is an example of what can be supported by LEO:

$$L=\prod_{cm=1}^{CM} \sum_{kcm=1}^{Kcm} PW_{kcm}(xbprodi(I)\prod_{i=1}^{I} \sum_{ki=1}^{Ki} PW_{ki}\prod_{t=1}^{T} ((xbtemp)(DISCRETE_{IT}|\mu_{kcm},\mu_{ki})(CONTINUOUS_{IT}|\mu_{kcm},\mu_{ki})))$$

Note: xbprodi and xbtemp will be explained below.

LEO can support the following extensions:

1. Sample selection bias: a subset of the equations are not estimated for some observations
2. Dependent variable of one equation appears on the RHS of another equation
3. One community (cluster)
4. One time period per individual
5. If one community, then community and individual correlations can become individual and time varying correlations

DATA

- LEO requires raw (ascii) data.
- Data must also be sorted by cluster, individual, and time period.
- The user must supply a constant variable, which can double as a time period and/or

cluster identifier if the data have only one time period and/or cluster.

STARTING VALUES FILE

The user must supply a file of starting values as raw (ascii) data. The best starting points for the starting values are the results of each equation run independently in Stata, SAS, or some other program. The values must be listed vertically and in order in the starting values file. The order is determined in the setup file.

The required order is as follows:

Logit equations

1. unconditional parameters
2. conditional parameters repeat for additional logit equations
3. continuous equation parameters
4. starting value for standard deviation of dependent variable (must be greater than 0)
5. repeat for additional continuous outcome equations
6. cluster level unobserved heterogeneity parameters for first comparison group of first logit equation
7. individual level unobserved heterogeneity parameters for first comparison group of first logit equation
8. cluster level unobserved heterogeneity parameters for second comparison group of first logit equation
9. individual level unobserved heterogeneity parameters for second comparison group of first logit equation, etc.
10. cluster level unobserved heterogeneity parameters for first comparison group of second logit equation
11. individual level unobserved heterogeneity parameters for first comparison group of second logit equation
12. cluster level unobserved heterogeneity parameters for second comparison group of second logit equation
13. individual level unobserved heterogeneity parameters for second comparison group of second logit equation, etc.
14. cluster level unobserved heterogeneity parameters for first continuous equation
15. individual level unobserved heterogeneity parameters for first continuous equation
16. cluster level unobserved heterogeneity parameters for second continuous equation
17. individual level unobserved heterogeneity parameters for second continuous equation, etc.
18. cluster level probability weight parameters (see below)
19. cluster level mass point parameters (see below)
20. individual level probability weight parameters (see below)
21. individual level mass point parameters (see below)

PROBABILITY WEIGHT AND MASS POINT PARAMETERS

If Kcl is the number of cluster level mass points specified, then there will be Kcl-1 probability weight parameters and Kcl-2 mass point parameters to be estimated.

The first mass point is set at 0. The last is set at 1, others are set at gamma, where gamma is the parameter to be estimated. Probability weight parameters are estimated for the 2...Kcl mass points.

The probability weight for the first mass point is equal to 1.

The probability weight for the other mass points is estimated.

NO ESTIMATION

LEO allows equation parameters to be "turned-off" for some subset of the estimation. That is, if so specified, certain parameters will not change from the starting values. This can be useful for two purposes. First, the noest (no estimation) feature can be used to exclude variables from the estimation. Second, it is sometimes necessary to keep masspoint parameters from changing until the model has "settled down" to some degree. For example: say you have estimated the model with three individual level mass points, and you want to add a fourth mass point. But when you try to estimate the new specification, mass point 3 and 4 converge to the same location. By preventing a change in the mass point locations until iteration 50, the two mass points might settle on different locations.

The noest file must include (in a column) as many numbers (0 or 1) as parameters in the model. If the number corresponding with a parameter is 0, the parameter is estimated. If the number is 1, the parameter will not be estimated. If the parameters are all to be estimated, then the file should simply contain a column of zeros.

ITERATION LIMITS

LEO can call OPT three times. First, the noest file is read. Then OPT(DFP) can be called.

Second, all the parameters are allowed to change and OPT(DFP) can be called again. Third, OPT(GRADX) can be called. The user specifies whether each call is made, and the iteration limit for each call.

The following logic is followed:

1. Is the iteration limit for the first CALL OPT(DFP) = 0? Yes, then go to 3.
2. If the iteration limit for the first CALL OPT(DFP) > O? Yes, then read noest file and CALL OPT(DFP).
3. Is the iteration limit for the second CALL OPT(DFP) = 0? Yes, then go to 5.
4. If the iteration limit for the second CALL OPT(DFP) > 0?

Yes, then allow all parameters to be estimated using OPT(DFP).

1. Is the iteration limit for the third CALL OPT(GRADX) = 0? Yes, then go to 7.
2. If the iteration limit for the second CALL OPT(GRADX) > 0? Yes, then CALL OPT(GRADX)
3. Done with OPT

LEO sends results based on OPTMOV to the results file after each call to OPT.

MAGNIFICATION OF VARIOUS CONTRIBUTIONS TO THE LIKELIHOOD FUNCTION

Under some circumstances, one or more of the elements of the likelihood function will become so large that the processor will round the value of the element to zero (i.e. numbers smaller than 1.0E-308). To circumvent this problem, LEO can magnify elements to the likelihood function. LEO can multiply observations' (time period level, not individual level see function above) contributions by a scaler and/or it can multiply community contributions by a multiple of a scaler (the multiple is based on the number of individuals (not observations) in the community. For example, say there is a community with 100 individuals and another with 50. It is possible to multiply each community's contribution by 1.0E+20 for every 10 individuals in the community. This results in a bigger multiplier for the larger community.

SETUP

LEO requires the user to provide the following four files.

Setup file: Must be called s.fil. The s.fil controls the estimation.

Data file: The data file is named in the s.fil (must be a raw or ascii file).

Starting values file: The starting values file holds all the initial parameter values.

No estimation file: The no estimation (noest) file contains a list of zeros and ones. A one turns off parameters so that they will not be changed by GQOPT.

LEO creates four additional files:

Results file

Ending values file: vector of ending parameters

CKPRS (check first partials) dump: output from CKPRS

GQOPT dump: output from GQOPT

The setup file (s.fil) is the file which sets up the parameters of the estimation. In it, files are named and equations, discrete factors, parameters of the estimation are specified.

The setup file must be set up as follows:

| 1. | | a80 | | read()title | |
| --- | --- | --- | --- | --- | --- |
| 2. | | a80 | | read()outfile | |
| 3. | | a80 | | read()datafile | |
| 4. | | a80 | | read()startfile | |
| 5. | | a80 | | read()noestfile | |
| 6. | | a80 | | read()ckfpdfile | |
| 7. | | a80 | | read()endfile | |
| 8. | | int | | read()iterla,iterlb,iterlc | |
| 9. | | int | | read()chunk | |
| 10. | | 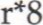 | | read()xbtemp,xbprodi,njump | |
| 11. | | int | | read()nvardata | |
| 12. | | int | | read()mlogit | |
| 13. | | int | | read()continuous | |
| 14. | | int | | read()mdvdata(mm),ncat(mm),nmvar(mm),nuncondv(mm), ncondv(mm) | |
| 15. | | int | | read()(mvardata(ll,mm),ll=l,nmvar(mm)) | |
| 16. | | int | | read()cdvdata(cc),ncvar(cc) | |
| 17. | | int | | read()(cvardata(ll,cc),ll=l,ncvar(cc)) | |
| 18. | | int | | read()cluster,ncl | |
| 19. | | int | | read()individual | |
| 20. | | int | | read()time,nt | |
| 21. | | int | | read()nmasscl,nmassi | |
| 22. | | int | | read()nxxvar | |
| 23. | | int | | read()(xxlist(jj),jj=l,nxxvar) | |
| 24. | | a8 | | read()(label(jj),jj=l,nvardata) | |

Explanations:

1. A title to appear at the top of the results file
2. Name (including path if necessary) of results file
3. Name (including path if necessary) of data file
4. Name (including path if necessary) of starting values file
5. Name (including path if necessary) of no estimation file
6. Name (including path if necessary) of ckprs output file
7. Name (including path if necessary) of ending values file
8. Three numbers are required. Iteration limit for each call to GQOPT. See Iteration Limits above.
9. Chunk is the number of clusters to be assigned to each proc during func, fp, and sp. This number must be less than or equal to nclus/nprocs or LEO will hang. Recommended: chunk=int(nclus/nprocs).
10. Three numbers are required. These numbers simply magnify a contribution to the likelihood function.

The first number magnifies an observation's contribution to the likelihood function (choose a number between 1.0d0 and 30.0d0).

The second number magnifies a cluster's contribution to the likelihood function. The second number will be multiplied to the cluster's contribution for every individual that is a multiple of the third number specified. For example, in a cluster with 45 individuals, if the second number is 1.0d20 and the third number is 10, then the cluster's contribution will be multiplied by 4*1.0d20.

1. The number of variables in the data set (the data set must include a constant
2. The number of discrete outcome equations
3. The number of continuous outcome equations

Note: a variable's "number label" is the number of the variable in the data set. If the variable with the character label "age" is the sixth variable in the data set, then the variable's number label is "6".

Logit equation specification

1. Five numbers are required.
   1. The number label of the dependent variable
   2. The number of categories in the dependent variable
   3. The number of right hand side (RHS) variables in the equation
   4. The number of RHS unconditional variables
   5. The number of RHS conditional variables (4+5=3)
2. List of the number labels of the variables in the equation (place unconditional variables before conditional variables, group unconditional variables by concept
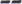
if price of each category is a concept, place all price variables together) repeat 14 and 15 for each logit equation

Continuous equation specification

1. Two numbers are required.
   1. Number label of dependent variable
   2. Number of variables on RHS of equation
2. List of the number labels of the variables in the equation

Repeat 16 and 17 for each continuous outcome equation

1. Two numbers required.
   1. Number label of community id variable
   2. Number of communities in data set
2. One number, Number label of individual id variable
3. Two numbers required.
   1. Number label of time period id variable
   2. Number of time periods covered by data set

Hint: use constant if only 1 community (in 18) use constant if only 1 time period (in 20)

1. Two Numbers required.
   1. number of community level mass points to be included (minimum—I)
   2. number of individual level mass points to be included (minimum=l) Note: 1 mass point means No correction.
2. Number of variables used on the RHS of the model (each counted only once)
3. List of variables (using number labels) used in the model (each used only once)
4. Character labels of variables in the data set (in order)

A note about second partials (SP) routine which is required for GRADX but not DFP. The SP accessed through the ENTRY SP requires more clusters than there are parameters to be estimated (otherwise the Hessian will be non-singular.

TIPS

Starting values: Try Stata coefficients, making them all 0.0 except for the constants and the SD of the error terms of the continuous equations.

Mass point (MP) strategies: Hold cluster MP to 1 and add individual MP one at a time until the number of points of improvement in the function value is less than the number of parameters added. Use the end.fil values as start values in each new run, except when increasing from 1 to 2 MP of each type. Then use 0.1 as the unobserved heterogeneity parameter start value. Use "x" in a=exp(x) to arrive at a starting value for the mass point parameters for #3 and over, putting "a" between the values with the largest weights from the previous run. Use 0.0 for the probability weight starting values. When convergence is reached, use the end values as starting values for the next run, dropping all individual MP and adding successive cluster MP until convergence.

SIMLEO

Simleo allows you to simulate various sample characteristics and observe changes in the outcome variables. Starting from the end.fil values from the best run of LEO, create the following files:

1. binary version of the raw data file (use convert to convert from raw to binary)
2. start.fil (use end.fil values unchanged)
3. probwt.fil copying the cluster MP probability weights, followed by the individual

MP probability weights from the Leo results file

1. noest.fil - same as LEO Create s.fil as follows:
2. Title (same as LEO)
3. Results file name (same as LEO)
4. Binary data file name
5. Start values file name
6. Probability weights file name
7. Noest file name (same as LEO)
8. Magic numbers (see LEO s.fil line #10)
9. Number of variables in the data file
10. Number of discrete outcome equations
11. Number of continuous outcome equations
12. Logit equation(s) specifications (same as LEO s.fil #14-15)
13. Continuous equation(s) specifications (same as LEO s.fil #16-17)
14. Community ID and number of communities (same as LEO s.fil #18)
15. Individual ID (same as LEO s.fil #19)
16. Four numbers are required.
    1. Number label of time period variable used in LEO (same as LEO s.fil #20)
    2. Number of time periods in above variable (same as LEO s.fil #20)
    3. Number label of actual time period variable (may be same as #1 above)
    4. Number of time periods in above variable (may be same as #2 above)
17. Mass points (same as Leo s.fil #21)
18. Number of RHS variables (same as LEO s.fil #22)
19. List of RHS variables (same as Leo s.fil #23)
20. Order in which equations will be calculated, on a single line:

Type "m" or "c" for each equation type, followed by the equation numbers as they are listed in the series of equation specifications (lines #11 & 12 above), e.g., for 2 mlogit and 1 continuous, with the continuous calculated second and the second mlogit calculated last: m cm 1 1 2

1. The number of variable substitutions to be made in the RHS vars in the last equation listed in line #19 (0 if no substitutions).
2. One line of 4 numbers is required for each variable substitution (if line #20 is greater than 0):
   1. Substitution type:
      1. = continuous outcome ==> mlogit RHS variable
      2. = mlogit outcome ==> mlogit RHS variables
      3. = continuous outcome ==> continuous RHS variable
      4. = mlogit outcome ==> continuous RHS variable
   2. Which RHS variable will be changed, where the number is the order in the model statement given in lines #11 & 12 above, not the order in the data.
   3. Which equation's DV will go into the RHS listed in #21-2, where the order is determined in line #19 above, e.g., if the continuous outcome will go into the RHS of the last equation in the example above, this value would be "2" because the continuous equation is listed second in line #19.
   4. Which mlogit outcome group will go into the RHS variable (use O for continuous equations)
3. Number of simulations to be run (0 if this is the base run with no simulations)
4. Two lines are required for each simulation (if #22 is greater than 0).
   1. The number (nl) of variables to be changed in the first simulation
   2. A vector of (nl) variable number labels, followed on the same line by a vector of (nl) values to which those variables will be changed for the first simulation repeat #23 for each simulation
5. Character labels of variables in the data file (same as Leo s.fil #24)

Simleo calculates the function value (blog), which should be close to the result from LEO if you run it without any simulation variables. It uses the probability weights to calculate the average of each individual's weighted probability of being in each mlogit outcome category, and the average of each individual's weighted value for each continuous outcome variable.

**Example Setup File for LEO**

Yearly runs

out-results.txt

data_fix_year.raw

end_het_3_2_may_18_9.txt

noest0.txt

end_het_3_2_may_18_10.txt

2 0 0

1. 1.0 10

47

3

0

7 2 14 14 0

6 34 11 12 18 13 25 26 21

22 19 20 43 4

9 2 15 15 0

6 34 11 12 18 13 25 26 21

22 19 20 16 44 4

10 5 15 15 0

6 11 12 18 13 21 22 19

20 16 31 32 40 42 4

5 155

2

3 13

4

3

2

47

1 2 3 4 5 6 7 8 9 10 11 12 13 14 15 16 17 18 19 20

21 22 23 24 25 26 27 28 29 30 31 32 33 34 35 36 37 38 39 40 41 42

43 44 45 46 47

personid double %10.0f 1

fake_id float %9.0g 2

time float %9.0g 3

one float %9.0g 4

location_code float %9.0g 5

ehage float %9.0g 6

first_sex float %9.0g 7

first_marr float %9.0g 8

ehbirth float %9.0g 9

method_year

eheduc_yrs float %9.0g 11

ilorin float %9.0g 12

muslim float %9.0g 13

sncl_timewait float %9.0g 14

sncl_idealfirst float %9.0g 15

age_first_sex float %9.0g 16

sncl_idealgap float %9.0g 17

jos float %9.0g 18

dur_resid_cit~1 float %9.0g 19

dur_resid_cit~2 float %9.0g 20

how_relig_2 float %9.0g 21

mus_how_relig_2 float %9.0g 22

sncl_bfapprFP float %9.0g 23

sncl_hearsee_~P float %9.0g 24

muslim_ilorin float %9.0g 25

muslim_jos float %9.0g 26

marr float %9.0g 27

age_first_mar~r float %9.0g 28

dur_until_mar float %9.0g 29

dur_until_mar~s float %9.0g 30

parity_1 float %9.0g 31

parity_2 float %9.0g 32

dur_until_adopt float %9.0g 33

ehage_sq

praise

com_praise

com_sex_act

com_unmarr_use

com_good_many_child

weighted_praise_indv_mean

weighted_provide_indv_mean

weighted_myths_indv_mean

weighted_SEX_indv_mean

weighted_birth_indv_mean

weighted_many_indv_mean

weighted_early_indv_mean

weighted_gap_indv_mean

**Fortran Code for Simulation Program (run in Fortran 77)**

IMPLICIT REAL*8 (A-H,O-Z)

DIMENSION X(6,155,150,13,20),TEMP(400),IREG(6),temps(400)

REAL*4 x,ran3,gasdev

integer*4 seed

DIMENSION XS(400),iord(6,40),cov(120,120),xncom(300),com(300)

DIMENSION TH(6,40),ran(6,60000),rdev(400),ch(160000),com2(300)

dimension hetc(6,10),heti(6,10),probc(10),probi(10),p(10)

DIMENSION XM(700),SDX(100),psum(6),xb(6),prob(10,10,10)

Dimension var(40000),u(400),e(400),cov_fact(40000),ncom(300)

dimension thmlogit(6,40),birth_indic(6,155,150,13),xb_mlogit(6)

dimension hetc_m(6,6),heti_m(6,6),probc_m(6),probi_m(6)

dimension prob_m(6,6,6),psum_m(6)

OPEN (15,FILE='setsim_2.fil')

OPEN (16,FILE='temp.res')

open (18,file='coef_nohet_july_9.txt')

open (19,file='pred_sex.raw')

open (20,file='simulation_13.raw')

open (21,file='cov_nohet_july_9.raw')

open(22,file='ncom.raw')

open (23,file='pred_birth.raw')

open (24,file='method.raw')

open (25,file='parity_check.raw')

READ (15,*) NVAR,NP,NEQ,(IREG(I),I=1,NEQ)

write (*,*) ' Nvar',nvar

write (*,*) ' NP',np

write (*,*) ' NEQ',neq

write (*,*) ' ireg',(ireg(i),i=1,neq)

idum=8154206

npts_c=3

npts_i=2

neq1=neq-1

ncat1=4

ncat=5

do j=1,NEQ

READ (15,*) (IORD(j,I),I=1,IREG(j))

enddo

read (18,*) (temps(i),i=1,np)

do i=1,NP

write (*,*) i

read (21,*) (cov(i,j),j=1,NP)

write (*,*) (cov(i,j),j=1,np)

enddo

c if (np .gt. 0) stop

do i=1,NEQ1

hetc(i,1)=0.d0

heti(i,1)=0.d0

enddo

probc(1)=1.0

probi(1)=1.0

do i=1,ncat1

hetc_m(i,1)=0.d0

heti_m(i,1)=0.d0

enddo

probc_m(1)=1.d0

probi_m(1)=1.d0

NOB=0

c if (nob .le. 0) stop

NC=155

ntime=13

DO I=1,NC

READ(22,*) NCOM(I)

xncom(i)=ncom(i)

ENDDO

do 100 i=1,NC

do 100 j=1,ncom(i)

do 100 k=1,ntime

c write (*,*) i,j,k

READ(20,*) (XS(II),II=1,NVAR)

c xs(13)=1.0

c xs(22)=xs(21)

c xs(25)=xs(12)

c xs(26)=xs(18)

c xs(42)=.9714

nob=nob+1

c write (*,*) nob

c if (nob .gt. 5) stop

do 160 jjj=1,NEQ

DO 160 JJ=1,IREG(jjj)

160 X(jjj,i,j,k,JJ)=XS(IORD(jjj,JJ))

100 CONTINUE

c IF (NOB .GT. 0) STOP

WRITE (16,7800)

7800 FORMAT (/,' MEANS AND SD ',/)

nob2=0

DO 140 I=1,IREG(3)

nob2=0

XM(I)=0.D0

SDX(I)=0.D0

DO 140 J=1,NC

do 140 k=1,ncom(j)

do 140 kk=1,ntime

nob2=nob2+1

XM(I)=XM(I)+X(3,j,k,kk,i)

140 SDX(I)=SDX(I) + X(3,J,k,kk,i)**2

WRITE (16,5203) NOB2

5203 FORMAT (' ACTUAL NUMBER OF OBSERVATIONS',I10,/)

XNOB=NOB

xnob2=nob2

DO 141 I=1,IREG(3)

XM(I)=XM(I)/XNOB2

SDX(I)=(SDX(I) -XNOB2*XM(I)**2)/(XNOB2-1.D0)

if (sdx(i) .le. .0001) sdx(i)=.0000001

c write (*,*) i,sdx(i)

SDX(I)=DSQRT(SDX(I))

141 WRITE (16,6500) I,XM(I),SDX(I)

6500 FORMAT (1X,I5,5X,5X,F12.4,5X,F12.4)

write (*,*) 'got to here'

c if (nob .gt. 0) stop

C STORE COVARANCE MATRIX AS A VECTOR

DO I=1,np

DO J=1,I

IJ=(((I-1)*I)/2)+J

var(IJ)=COV(I,J)

write (*,*)var(ij)

ENDDO

ENDDO

C USE SUBROUTINE CHOLES TO GET COV_FACT -- LOWER TRIANGLE MATRIX

call choles (var,40000,100,COV_FACT)

idum=-7654321

do 470 ibs=1,1000

c do 470 ibs=1,1

write (*,*) ibs

do j=1,np

U(j)=gasdev(idum)

enddo

C GENERATE MULTIVARIATE NORMAL ERRORS

call errget(E,100,COV_FACT,40000,U)

do isf=1,NP

c write (*,*) rdev(isf)

c rdev(isf)=0.d0

c temp(isf)=temps(isf)+e(isf)

temp(isf)=temps(isf)

enddo

icount=0

DO I=1,neq1

do j=1,ireg(I)

icount=icount+1

TH(I,j)=TEMP(Icount)

write (16,*) icount, i,j,th(i,j)

ENDDO

enddo

do i=1,ncat1

do j=1,ireg(3)

icount=icount+1

thmlogit(i,j)=temp(icount)

write (16,*) icount, i,j,thmlogit(i,j)

enddo

enddo

do 502 ineq=1,neq1

if (npts_c .eq. 1) go to 501

cc icount=35

do i=2,npts_c

icount=icount+1

hetc(ineq,i)=temp(Icount)

write (16,*) 'hetc', ineq,i,hetc(ineq,i)

enddo

501 if (npts_i .eq. 1) go to 502

do i=2,npts_i

icount=icount+1

heti(ineq,i)=temp(icount)

write (16,*) 'heti',ineq,i,heti(ineq,i)

enddo

502 continue

if (npts_c .eq. 1) go to 531

do 560 ineq=1,ncat1

cc icount=35

do i=2,npts_c

icount=icount+1

hetc_m(ineq,i)=temp(Icount)

write (16,*) 'hetc_m',icount, ineq,i,hetc_m(ineq,i)

enddo

560 continue

531 if (npts_i .eq. 1) go to 532

do 561 ineq=1,ncat1

do i=2,npts_i

icount=icount+1

heti_m(ineq,i)=temp(icount)

write (16,*) 'heti_m',icount,ineq,i,heti_m(ineq,i)

enddo

561 continue

532 continue

if (npts_c .eq. 1) go to 504

pc=1.d0

icount_t=icount

c icount=83

do i=2,npts_c

icount_t=icount_t+1

pc=pc+dexp(temp(icount_t))

enddo

probc(1)=1.d0/pc

do i=2,npts_c

icount=icount+1

probc(i)=dexp(temp(icount))/pc

enddo

do i=1,npts_c

write (16,*) 'probc',i,probc(i)

enddo

504 if (npts_i .eq.1) go to 505

pi=1.d0

icount_t=icount

do i=2,npts_i

icount_t=icount_t+1

pi=pi+dexp(temp(icount_t))

enddo

probi(1)=1.d0/pi

do i=2,npts_i

icount=icount+1

probi(i)=dexp(temp(icount))/pi

enddo

do i=1,npts_i

write (16,*) 'probi',i,probi(i)

enddo

505 continue

c if (i .gt. 0) stop

do i=1,nc

do j=1,ncom(i)

do k=1,ntime

x(2,i,j,k,10)=-10.d0

x(3,i,j,k,10)=-10.d0

x(3,i,j,k,11)=-10.d0

enddo

enddo

enddo

do 508 nq=1,1

do 506 i=1,NC

do 506 j=1,ncom(i)

psave=1.d0

age_sex=0.d0

do 507 k=1,ntime

c write (19,*) i,j,k

xb(nq)=0.d0

do jj=1,ireg(nq)

xb(nq)=xb(nq)+x(nq,i,j,k,jj)*th(nq,jj)

c write (*,*) i,j,k,jj,th(nq,jj),x(nq,i,j,k,jj),xb(nq)

c if (j .gt. 2) stop

enddo

psum(nq)=0.d0

do ip=1,npts_c

do jp=1,npts_i

xbh=xb(nq)+hetc(nq,ip)+heti(nq,jp)

prob(nq,ip,jp)=dexp(xbh)/(1.d0+dexp(xbh))

psum(nq)=psum(nq)+probc(ip)*probi(jp)*prob(nq,ip,jp)

enddo

enddo

check=ran3(idum)

prob_sex=psum(nq)

c prob_sex=psum(nq)*psave

c write (19,*) i,j,k,xb(nq),xbh,check,psum(nq),psave

if (check .gt. prob_sex) go to 200

age_sex=k+11

c if( k .lt. 8) go to 507

c age_sex=19

x(2,i,j,k,10)=age_sex

c if (k.eq. 8) go to 510

go to 510

200 continue

c 200 psave=psave*(1.d0-psum(nq))

507 continue

510 continue

write(19,*) ibs,i,j,k,age_sex

506 continue

508 continue

do i=1,nc

do j=1,ncom(i)

do k=1,ntime

if (k .eq. 1) go to 216

if (x(2,i,j,k-1,10) .gt. 0.d0) x(2,i,j,k,10)=x(2,i,j,k-1,10)

216 continue

x(3,i,j,k,9)=x(2,i,j,k,10)

enddo

enddo

enddo

do 518 nq=2,2

do 516 i=1,NC

do 516 j=1,ncom(i)

psave=1.d0

births=0.d0

do 517 k=1,ntime

birth_indic(3,i,j,k)=0.d0

if (x(2,i,j,k,10) .lt. 0) go to 517

c write (19,*) i,j,k

xb(nq)=0.d0

do jj=1,ireg(nq)

xb(nq)=xb(nq)+x(nq,i,j,k,jj)*th(nq,jj)

c write (*,*) i,j,k,jj,th(nq,jj),x(nq,i,j,k,jj),xb(nq)

c if (j .gt. 2) stop

enddo

psum(nq)=0.d0

do ip=1,npts_c

do jp=1,npts_i

xbh=xb(nq)+hetc(nq,ip)+heti(nq,jp)

prob(nq,ip,jp)=dexp(xbh)/(1.d0+dexp(xbh))

psum(nq)=psum(nq)+probc(ip)*probi(jp)*prob(nq,ip,jp)

enddo

enddo

check=ran3(idum)

prob_birth=psum(nq)

c prob_sex=psum(nq)*psave

c write (19,*) i,j,k,xb(nq),xbh,check,psum(nq),psave

if (check .gt. prob_birth) go to 210

births=births+1.d0

birth_indic(3,i,j,k)=1.d0

x(3,i,j,k,10)=0.d0

if (births .eq. 1.d0) x(3,i,j,k,10)=1.d0

x(3,i,j,k,11)=0.d0

if(births .eq. 2.d0) x(3,i,j,k,11)=1.d0

write (25,*) ibs,i,j,k,x(3,i,j,k,10),x(3,i,j,k,11)

go to 517

210 continue

c 200 psave=psave*(1.d0-psum(nq))

517 continue

write(23,*) ibs, i,j,k,births

516 continue

518 continue

do 528 nq=3.3

do 526 i=1,NC

do 526 j=1,ncom(i)

do 527 k=1,ntime

if (birth_indic(3,i,j,k) .eq. 0.d0) go to 527

c write (19,*) i,j,k

do kk=1,ncat1

xb_mlogit(kk)=0.d0

do jj=1,ireg(nq)

xb_mlogit(kk)=xb_mlogit(kk)+x(nq,i,j,k,jj)*thmlogit(kk,jj)

c write (*,*) i,j,k,kk,jj,thmlogit(kk,jj),x(3,i,j,k,jj)

c 1 ,xb_mlogit(kk)

c if (j .gt. 5) stop

enddo

c write (*,*) i,j,k,kk,xb_mlogit(kk)

c if (j .gt. 2) stop

enddo

do kk=1,ncat

psum_m(kk)=0.d0

enddo

do ip=1,npts_c

do jp=1,npts_i

ptemp=1.d0

do kk=1,ncat1

xbh=xb_mlogit(kk)+hetc_m(kk,ip)+heti_m(kk,jp)

c write (*,*) xbh

prob_m(kk+1,ip,jp)=dexp(xbh)

ptemp=ptemp+prob_m(kk+1,ip,jp)

enddo

prob_m(1,ip,jp)=1.d0

do kk=1,ncat

prob_m(kk,ip,jp)=prob_m(kk,ip,jp)/ptemp

c write (*,*) kk, ptemp, prob_m(kk,ip,jp)

enddo

do kk=1,ncat

psum_m(kk)=psum_m(kk)+probc(ip)*probi(jp)*prob_m(kk,ip,jp)

enddo

enddo

enddo

c write (*,*) i,j,k,(psum_m(kk),kk=1,5)

c if (j .gt. 10) stop

temp1=psum_m(1)

temp12=psum_m(1)+psum_m(2)

temp123=psum_m(1)+psum_m(2)+psum_m(3)

temp1234=psum_m(1)+psum_m(2)+psum_m(3)+psum_m(4)

do kk=1,ncat

psum_m(kk)=0.d0

enddo

check=ran3(idum)

if(check .le. temp1) psum_m(1)=1.d0

if (check .gt. temp1 .and. check .le. temp12) psum_m(2)=1.d0

if (check .gt. temp12 .and. check .le. temp123) psum_m(3)=1.d0

if (check .gt. temp123 .and. check .le. temp1234) psum_m(4)=1.d0

if (check .gt. temp1234) psum_m(5)=1.d0

write(24,*) ibs,i,j,k,(psum_m(kk),kk=1,5),x(3,i,j,k,10)

1 ,x(3,i,j,k,11)

527 continue

526 continue

528 continue

470 continue

STOP

END

**References**

49. Mroz TA. Discrete factor approximations in simultaneous equation models: Estimating the impact of a dummy endogenous variable on a continuous outcome. Journal of Econometrics. 1999; 92(2): 233–274.
