# Supplementary material for: The direct and indirect effects of community beliefs and attitudes on postpartum contraceptive method choice among young women ages 15–24 in Nigeria
Source: PLoS One. 2022 Jan 27;17(1):e0261701. doi: 10.1371/journal.pone.0261701 (PMC8794167; doi:10.1371/journal.pone.0261701)
Supplement: Attachment — Submitted filename: PLOS Response to reviewers_Dec 2021_submit.docx [file pone.0261701.s004.docx]

Reviewer comments :

Thank you for submitting your manuscript to PLOS ONE. After careful consideration, we feel that it has merit but does not fully meet PLOS ONE’s publication criteria as it currently stands. Therefore, we invite you to submit a revised version of the manuscript that addresses the points raised during the review process.

The manuscript has considerably strengthened and is much clearer for a broad general audience such as PLOS ONE's. Congratulations to the authors. It is not felt necessary to send the article back to the reviewers.

There are, however, some details that are left unspecified and should be addressed according to PLOS ONE statistical guidelines. You mention in the reply that you are using non-standard software but no mention is given in the text, or any links to code for reproducibility. These are the specific PLOS ONE guidelines on the topic:

"In the methods, include a section on statistical analysis that reports a detailed description of the statistical methods. In this section:

- List the name and version of any software package used, alongside any relevant references
- Describe technical details or procedures required to reproduce the analysis
- Provide the repository identifier for any code used in the analysis (See our [code-sharing policy](https://journals.plos.org/plosone/s/materials-software-and-code-sharing).)"

Please update.

RESPONSE:

We have added relevant information into the statistical methods section on the estimation approaches and software used. In addition, we have added an additional supporting information file (S2) that includes the code for reproducing the analysis in Fortran. As we elaborate in the supporting information file, a total of three programs were used:

1. The Discrete Factor Approximation Method (DFAM)

This program estimates systems of equations with a mix of continuous and discrete dependent variables using semi-parametric maximum likelihood. It was written and copyrighted by Jeffrey Rous from the Department of Economics at North Texas State University. The manual for the package (referred to as LEO) describes the program, specifies the likelihood function and provides detailed instructions on how to write a setup file that the program uses to specify the set of equations and read in the raw data as an ascii file. A copy of the manual has been put included as part of the supporting information file (S2). The actual DFAM program code would have to be obtained from Jeffrey Rous and we have added this note to the text.

2. GQOPT

This is the Goldfeld-Quandt non-linear optimization package ([www.quandt.com/gqopt.html](http://www.quandt.com/gqopt.html)) that is marketed by Richard Quandt from Princeton University. The package includes a large number of non-linear optimization packages. The DFAM (LEO) program can invoke two of the packages: the Davidon-Fletcher-Powell (DFP) algorithm and the quadratic hill climbing algorithm (GRADX). We used DFP as a first step and then switched to GRADX when we got close to the maximum of the likelihood function to get more precision and a better estimate of the covariance matrix of the parameter estimates. In both cases, we used the default versions of the algorithms; this is described in the text and supporting information file (S2).

3. Simulation Program

The simulation program was written in Fortran 77 by one of the co-authors of this article and was specifically written to do the simulations described in detail in the text and the supporting information files (S1 and S2).

DFAM (LEO) was provided to us by Rous under the provision that we not share his copyrighted code. We refer readers to Rous at North Texas State University to obtain a copy. A site license for GQOPT was purchased by the Department of Economics at the University of North Carolina at Chapel Hill for use by our faculty and graduate students. We refer readers to Quandt to obtain a copy of the code. The simulation program (simulation.f) and the setup file for DFAM (s.fil) which were written by one of the co-authors of this article are included in supporting information file S2.

Additional Reviewer Comments:

Regarding interpretation of results,  and details regarding the description of results I would ask for some clarifications:

1. Please clarify on the time unit of analysis. Is it monthly and annual? It seems that the calendar data is monthly but at some time it is said that age is t+13 suggesting estimation is based on annual data.

This analysis was performed annually such that we examined use in the one year following a birth and examined the most effective method used in that year. Therefore, each woman contributes as many observations as births that she has in the calendar period. This has been clarified in the text.

2. How is method use defined in the presence of concurrent method use? Did you contemplate an alternative state for concurrent method use? Do you use the most effective method à la DHS?

In the calendar, only one method can be reported per month; interviewers were instructed to report the most effective method in that month if multiple methods were reported. This has been added to the text.

3. Related to that, if estimation is carried out with yearly and not monthly data: How do you define the method used in a year time? Any use? Most frequent method?

Most people only listed one method in the year postpartum. For the small number of women who used multiple methods in the year, we used the most effective method listed in the period. We consider long-acting methods the most effective followed by short acting methods. This has been clarified in the text.

4. This is particularly relevant in the case of LAM.

The order for effectiveness was none, traditional, LAM, short acting, long acting. If they simply reported LAM, then that was the method that they were assigned in the postpartum period. However, if they switched to a long-acting or short acting method during the year, then they were assigned that method rather than LAM. There are only 54 individuals that reported uniquely LAM use. Note that this use is coded based on use of a method (the most effective method) at some point during the year postpartum; all of the LAM use started immediately postpartum.

Maybe you could clarify that the woman might be using LAM without any intention to contracept. To the extent that the conditions of not menstruating, baby less than 6 months, and full breast-feeding are met the woman is using LAM. In this sense it is very different from the other methods (but you are allowing for this. You could comment in the manuscript).

We have added information into the text that we only coded LAM use if the woman reported that this was the method she was using to avoid a pregnancy and not based on the conditions listed above.

- Also, you cannot be using LAM for more than 6 months. It is therefore not a solution as a post-partum method (just for the immediate postpartum). I would like to know how this is captured in the model, particularly in connection with questions 2 and 3.

As mentioned above, LAM was only coded as the postpartum method if it was the only method listed in the one-year postpartum period; any woman who reported LAM and then another method after LAM that is more effective, would be coded based on the more effective method. Only 54 women who had a birth reported only LAM as their method in the postpartum period and all of this use started immediately postpartum.
